# The effects of T-DXd on the expression of HLA class I and chemokines CXCL9/10/11 in HER2-overexpressing gastric cancer cells

**DOI:** 10.1038/s41598-021-96521-2

**Published:** 2021-08-19

**Authors:** Shotaro Nakajima, Kosaku Mimura, Takuro Matsumoto, Aung Kyi Thar Min, Misato Ito, Hiroshi Nakano, Prajwal Neupane, Yasuyuki Kanke, Hirokazu Okayama, Motonobu Saito, Tomoyuki Momma, Yohei Watanabe, Hiroyuki Hanayama, Suguru Hayase, Zenichiro Saze, Koji Kono

**Affiliations:** 1 Department of Gastrointestinal Tract Surgery, Fukushima Medical University School of Medicine, 1 Hikariga-oka, Fukushima City, Fukushima 960-1295 Japan; 2 Department of Obesity and Inflammation Research, Fukushima Medical University School of Medicine, Fukushima, Japan; 3 Department of Blood Transfusion and Transplantation Immunology, Fukushima Medical University School of Medicine, Fukushima, Japan

**Keywords:** Tumour immunology, Gastrointestinal cancer

## Abstract

Trastuzumab deruxtecan (T-DXd), a HER2-targeting antibody–drug conjugate with a topoisomerase I inhibitor deruxtecan (DXd), exhibits an excellent anti-tumor effect in previously treated HER2-positive tumors. A recent study demonstrated that T-DXd not only suppressed tumor growth but also enhanced anti-tumor immunity through increasing the number of tumor-infiltrating CD8^+^ T cells and enhancement of major-histocompatibility-complex class I expression on tumor cells in a mouse model. However, the effect of T-DXd on anti-tumor immune responses in human cancers is largely unknown. We investigated the effect of T-DXd on the expression of HLA class I and CXCL9/10/11, T-cell chemoattractants, in HER2-positive human gastric cancer (GC) cells. We found that T-DXd significantly inhibited GC cell proliferation in a HER2-dependent manner, while it slightly increased the expression of HLA class I in HER2-positive GC cells. Moreover, we revealed that T-DXd significantly induced mRNA expression of *CXCL9/10/11* in HER2-positive GC cells. T-DXd-triggered up-regulation of these chemokines was mediated through the activation of DNA damage signaling pathways. These results suggest that T-DXd triggers anti-tumor immune responses at least in part through induction of the expression of HLA class I and *CXCL9/10/11* on HER2-positive GC cells, resulting in the enhancement of anti-tumor immunity in human GC.

## Introduction

Trastuzumab deruxtecan (T-DXd) is a HER2-targeting antibody–drug conjugate with a topoisomerase I inhibitor exatecan derivative (DX-8951 derivative, DXd)^1^. A humanized, monoclonal anti-HER2 antibody and DXd are combined by a tetrapeptide-based linker that can be cleaved by lysosomal enzymes in cancer cells after internalization^2^. Ogitani et al. demonstrated that T-DXd exhibited a great potential to inhibit the growth of low HER2-expressing and trastuzumab- or trastuzumab emtansine (T-DM1)-insensitive HER2-positive cancer cells in patient-derived xenograft models^1,3^. Indeed, in DESTINY-Breast01 clinical trial enrolling 184 female patients with unresectable or metastatic HER2-positive breast cancer who had received two or more prior anti-HER2-based regimens, T-DXd (5.4 mg/kg dose) showed favorable and durable anti-tumor activity^4^. Moreover, T-DXd (6.4 mg/kg dose) also led to significant improvements in response and overall survival among patients with HER2-positive gastric or gastroesophageal junction adenocarcinoma who had received at least two previous therapies including trastuzumab^5^. Interestingly, a previous report suggested that T-DXd enhanced anti-tumor immunity through tumor recognition by T cells in a mouse model^6^. The increment of tumor-infiltrating dendritic cells and CD8^+^ T cells, the augmentation of major-histocompatibility-complex (MHC) class I in cancer cells, and the rejection of rechallenged cancer cells by adaptive immune T cells were observed in an immunocompetent mouse model with human HER2-expressing murine colorectal cancer (CRC) cells, which might a reason for a superior anti-tumor effect of T-DXd as compared with trastuzumab alone and T-DM1. However, the effect of T-DXd in anti-tumor immune responses in human tumors is largely unknown.

Decreased expression of human leukocyte antigen (HLA) class I (human MHC class I) is often associated with disease progression and poor prognosis in several tumors due to low sensitivity to cancer cell lysis by cytotoxic T lymphocytes^7–11^. Our previous studies showed inverse correlations between HER2 expression and HLA class I expression in several human tumors including melanoma, carcinoma, breast cancer, esophageal cancer, and gastric cancer (GC)^12–15^. We also demonstrated that the activity of MAPK/ERK, an important downstream molecule of HER2-signaling, was inversely associated with the level of HLA class I expression in esophageal cancer and GC^15^. Therefore, blockade of HER2 signaling by T-DXd possibly contributes to the upregulation of HLA class I expression in HER2-positive GC cells. In addition, DXd, the payload of T-DXd, is a DNA topoisomerase I inhibitor, and topoisomerase I inhibitors cause DNA damage to cancer cells through blockade of the ligation step during replication of cell cycle, which generates DNA single- or double-strand breaks^16^. Interestingly, previous reports indicated that topoisomerase I inhibition triggered the activation of DNA damage signaling leading to enhanced expression of MHC class I in cancer cells, which may assist the immune system to eliminate these cells^17–19^. Because T-DXd, but not anti-HER2 antibody, has been reported to cause the activation of DNA damage signaling in HER2-positive cancer cells^1^, there is a possibility that T-DXd also can increase the expression of HLA class I through the activation of DNA damage signaling.

C–X–C motif chemokine ligand (CXCL)9/10/11 are known as T cell chemoattractants, which recruit anti-tumor cytotoxic T lymphocytes via its receptor C–X–C chemokine receptor 3 and inhibit tumor progression^20–22^. CXCL9/10/11 are synthesized and released from leukocytes, as well as epithelial, endothelial, and stromal cells, and the production of these chemokines is predominantly regulated by interferon-γ (IFN-γ) stimulation^23^. However, previous reports suggested that anti-cancer drugs also have the potential to induce CXCL9/10/11 release from cancer cells, and promote anti-tumor effects^24–26^. Cell cycle-specific DNA damage by cisplatin or hydroxyurea up-regulated mRNA expression of *CXCL9, -10* in human cervical cancer cells, breast cancer cells, and melanoma cells^26,27^. Because T-DXd, especially DXd has been reported to cause cell cycle-specific DNA damage in HER2-positive cancer cells, it possibly up-regulates the expression of CXCL9/10/11 through the induction of cell cycle-specific DNA damage in HER2-positive GC cells.

In this study, we investigated the effect of T-DXd on the expression of HLA class I and CXCL9/10/11 in HER2-positive GC cells. We also examined the underlying mechanism of how T-DXd regulated mRNA expression of *CXCL9/10/11* in HER2-positive GC cells.

## Results

### HER2-dependent inhibition of GC cell proliferation by T-DXd

**Figure 1:**
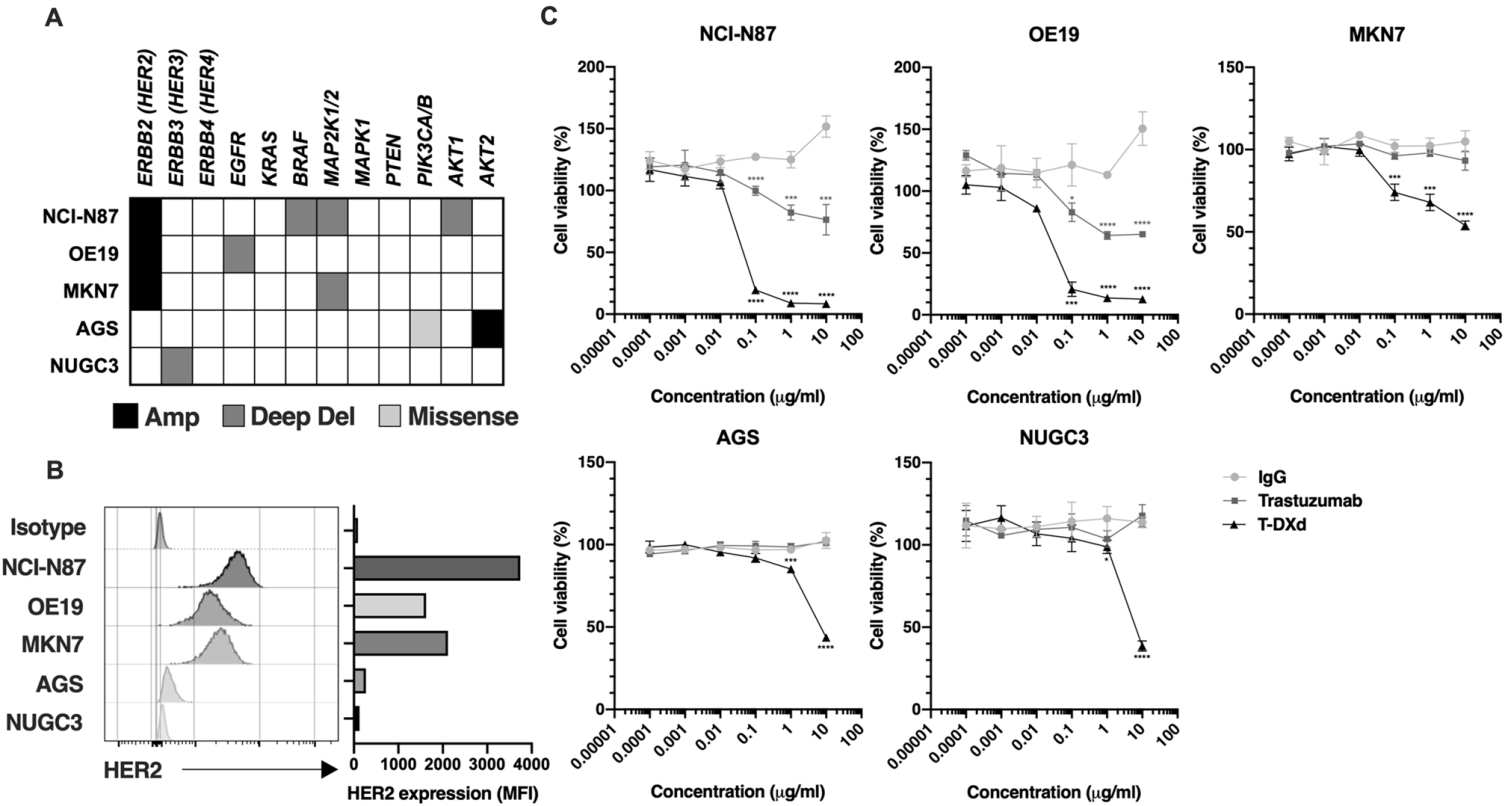
The effect of T-DXd on HER2-positive and HER2-negative GC cells. (**A**) Genetic alterations and mutations of the ErbB (HER) family and its downstream molecules in indicated GC cell lines. Black: amplification, gray: deep deletion, light gray: missense mutation, white: no alteration and no mutation. (**B**) HER2 expression on HER2-positive and HER2-negative GC cell lines. (**C**) Cell viability assay in HER2-positive and HER2-negative GC cell lines treated with several concentrations of human IgG isotype control, trastuzumab, or T-DXd for 6 days (n = 3). Values are shown as means ± SEM. *P < 0.05, ***P < 0.001, ****P < 0.0001.

To first assess the effect of T-DXd on cell proliferation of HER2-positive and HER2-negative GC cells, three HER2-amplified GC cell lines (NCI-N87, OE19, and MKN7) and two HER2-non-amplified GC cell lines (AGS and NUGC3) were used in this study (Fig. 1A). Although deep deletions (Deep del) and missense mutations (Missense) in the molecules of HER2 signaling including MAPK and AKT signaling pathways have been reported in the five GC cell lines, their biological significances (oncogenicities) are unknown (Fig. 1A). Cell surface overexpression of HER2 was confirmed by flow cytometry in NCI-N87, OE19, and MKN7 cells but not in AGS and NUGC3 cells (Fig. 1B). Using these five GC cell lines, we tested cell growth inhibitory activity by T-DXd in vitro. As shown in Fig. 1C, concentrations of more than 0.1 μg/ml T-DXd significantly suppressed cell proliferation of HER2-positive NCI-N87 and OE19 cells, and even in MKN7 cells, which are known as trastuzumab-resistant HER2-positive GC cells. On the other hand, T-DXd did not inhibit cell proliferation of HER2-negative AGS and NUGC3 cells (Fig. 1C). However, 10 μg/ml T-DXd markedly suppressed cell proliferation in both HER2-positive and HER2-negative GC cells (Fig. 1C). A previous report demonstrated that the higher concentration (10 μg/ml) of control IgG-ADC-conjugated with DXd had cell growth inhibition activity in several cancer cell lines, and the antibody-independent cytotoxicity occurred at higher concentrations of ADC-conjugated with DXd^28^. Based on the previous report and our present result, we strongly suggested that HER2-independent cell growth inhibition might have occurred in HER2-negative GC cell lines treated with 10 μg/ml T-DXd.

### Up-regulation of HLA class I expression by T-DXd in HER2-positive GC cells

**Figure 2:**
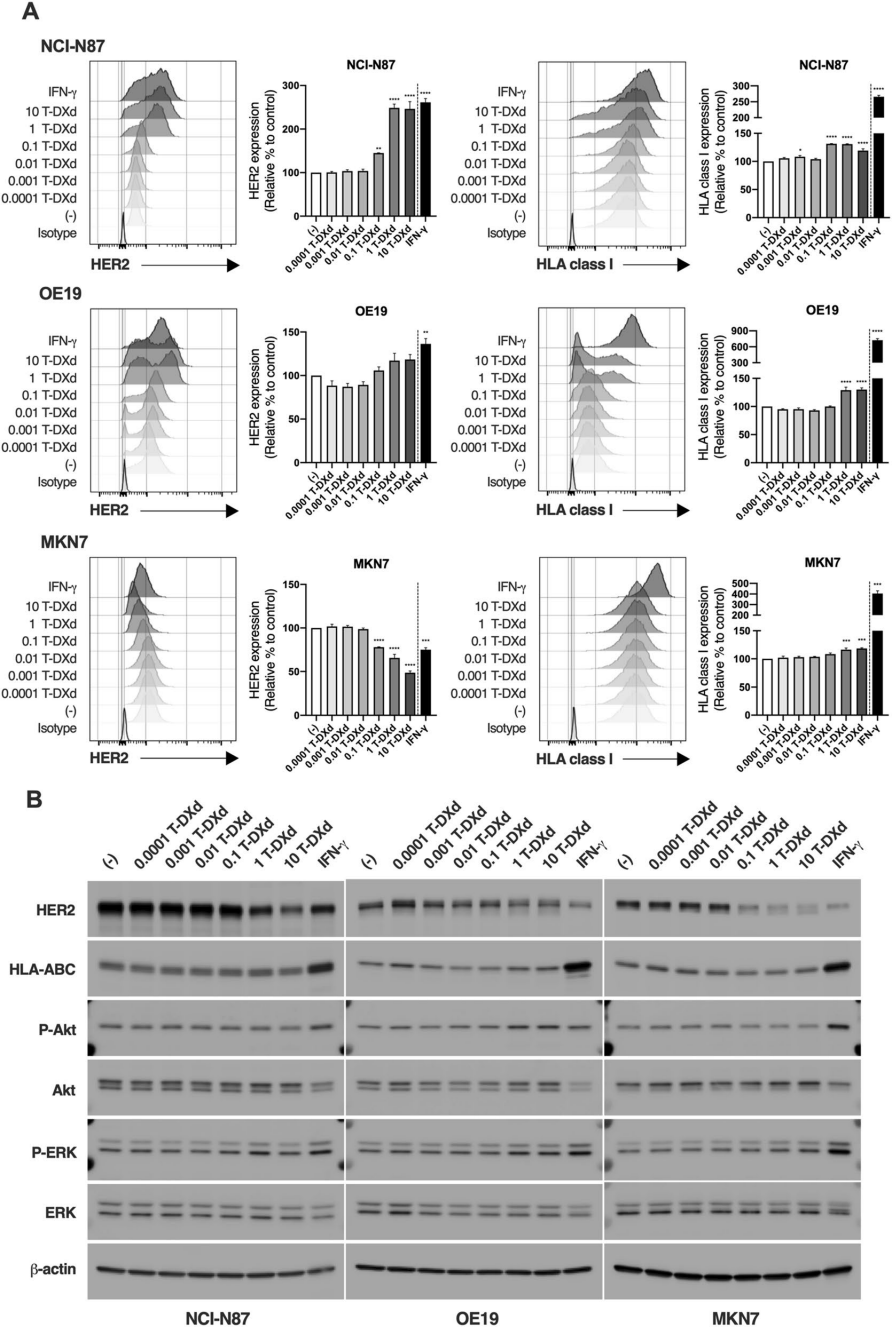
The effect of T-DXd on the expression of HLA class I in HER2-positive GC cells. (**A**) The expression of HER2 (left) and HLA class I (right) in HER2-positive GC cell lines treated with several concentrations of T-DXd or 100 ng/ml IFN-γ for 72 h (n = 3). Representative histograms were shown. Values are shown as means ± SEM. *P < 0.05, **P < 0.01, ***P < 0.001, ****P < 0.0001. (**B**) Western blot analysis of the indicated molecules in HER2-positive GC cell lines treated with several concentrations of T-DXd for 72 h.

To next examine the effect of T-DXd on the expression of HLA class I in HER2-positive GC cells, NCI-N87, OE-19, and MKN7 cells were treated with several concentrations of T-DXd and subjected to flow cytometry and western blot of HLA class I as well as HER2. The cell surface expression of HER2 was increased in NCI-N87 and OE19 cells by T-DXd, but not trastuzumab (Fig. 2A and Supplementary Fig. S1), while it was dramatically decreased by T-DXd and trastuzumab in MKN7 cells (Fig. 2A and Supplementary Fig. S1), suggesting that decreasing cell surface expression of HER2 might be one of the main mechanisms of resistance to trastuzumab in MKN7 cells due to low binding rate of trastuzumab to the cells. Although IFN-γ markedly increased both cell surface and protein expressions of HLA class I in HER2-positive GC cells (Fig. 2A,B), T-DXd caused a slight increase of cell surface expression of HLA class I (Fig. 2A). Because increased expression of HLA class I by T-DXd was not observed by western blot analysis (Fig. 2B), T-DXd might increase cell surface expression of HLA class I at the post-translational level in HER2-positive GC cells. Given that, (1) trastuzumab did not affect the expression of HLA class I in HER2-positive GC cells (Supplementary Fig. S1), (2) the levels of phosphorylated Akt and ERK were not obviously changed by the treatment with T-DXd, and (3) T-DXd moderately increased cell surface expression of HLA class I in HER2-positive GC cells regardless of genetic alterations and mutations of HER2 signaling molecules including *BRAF*, *MAP2K1/2,* and *AKT1* (Figs. 1A and 2A), the blockade of HER2 signaling pathways by T-DXd might not be crucial for the regulation of cell surface expression of HLA class I in HER2-positive GC cells. Indeed, the DNA topoisomerase I inhibitor, irinotecan, significantly increased cell surface expression of HLA class I in HER2-positive GC cells (Supplementary Fig. S2). These results suggest that T-DXd slightly increases cell surface expression of HLA class I in HER2-positive GC cells via the effect of DXd.

We also examined the effect of T-DXd on cell surface expression of HER2 and HLA class I in HER2-negative NUGC3 and AGS cells. As a result, the relative percentages of HER2 expression were slightly increased at 10 μg/ml T-DXd in NUGC3 cells or decreased at 0.1–10 μg/ml T-DXd in AGS cells (Supplementary Fig. S3, left). However, because the basal expression levels of HER2 were very low in both cell lines, T-DXd might have little effect on HER2 expression in NUGC3 and AGS cells. In addition, the expression of HLA class I in AGS cells was not increased by T-DXd, while only 10 μg/ml T-DXd slightly increased cell surface expression of HLA class I in NUGC3 cells (Supplementary Fig. S3, right), which might depend on HER2-independent cytotoxic effects of T-DXd. Because a previous report suggested that AGS cells were defective in MHC class I inducibility compared with other human cell lines^29^, the different expression of HLA class I by T-DXd might be observed between the two cell lines.

### Induction of mRNA expression of T-cell chemoattractants CXCL9/10/11 by T-DXd

**Figure 3:**
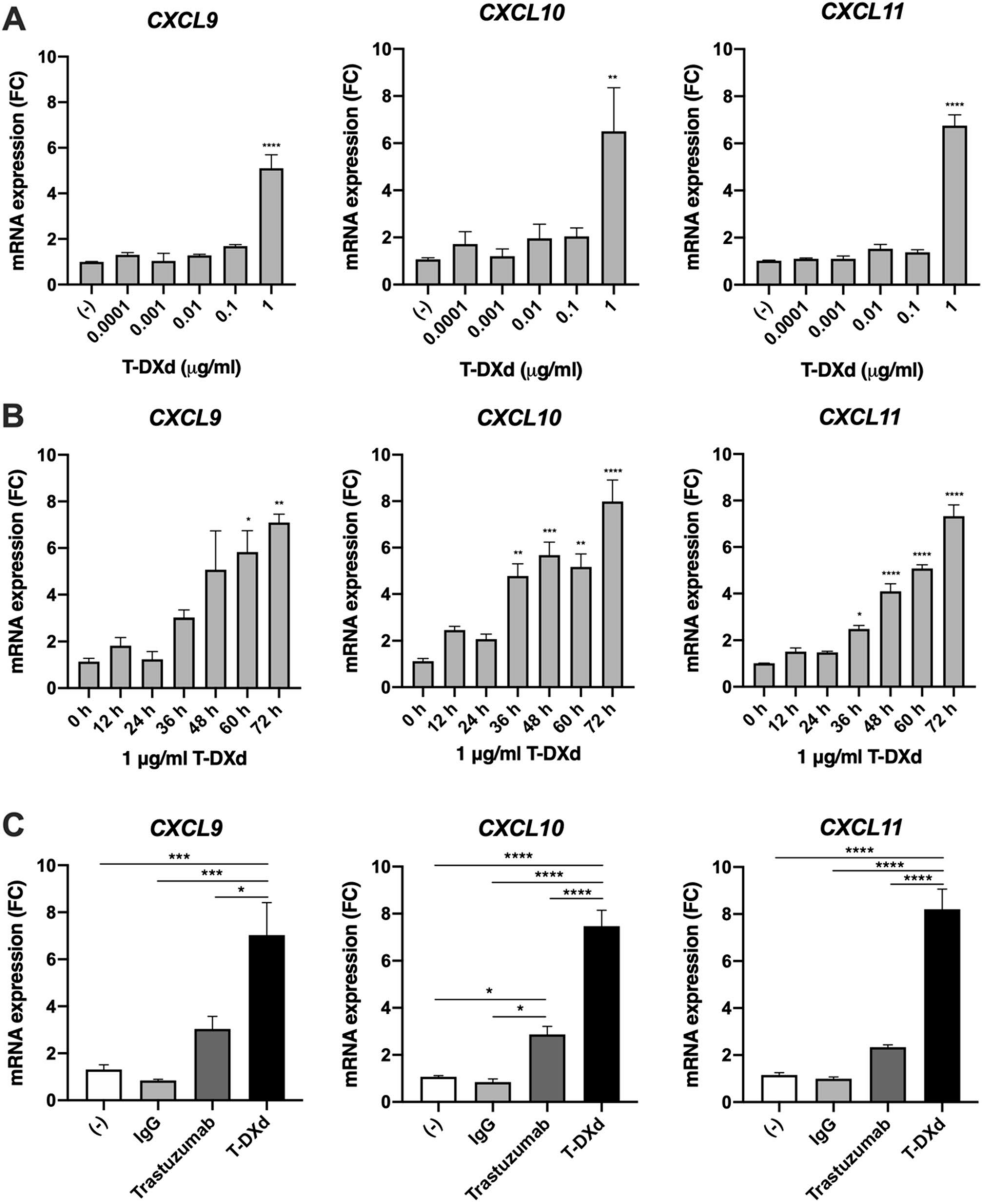
The effect of T-DXd on mRNA expression of *CXCL9/10/11* in HER2-positive GC cells. (**A**, **B**) Dose- and time-dependent effect of T-DXd on mRNA expression of *CXCL9*, *CXCL10,* and *CXCL11* in NCI-N87 cells (n = 3). (**C**) mRNA expression of *CXCL9*, *CXCL10,* and *CXCL11* in NCI-N87 cells treated with 1 μg/ml human IgG, trastuzumab, or T-DXd (n = 3). Values are shown as means ± SEM. *P < 0.05, ***P < 0.001, ****P < 0.0001.

In the previous report, T-DXd increased the number of tumor-infiltrating CD8^+^ T cells in a mouse model^6^. We, therefore, tested whether T-DXd triggered the expression of T-cell chemoattractants CXCL9/10/11 in HER2-positive GC cells. T-DXd significantly induced mRNA expression of *CXCL9/10/11* in a dose- and time-dependent manner in NCI–N87 cells (Fig. 3A,B). We found that mRNA expression of *CXCL9/10/11* induced by T-DXd was significantly higher than that induced by Trastuzumab (Fig. 3C).

**Figure 4:**
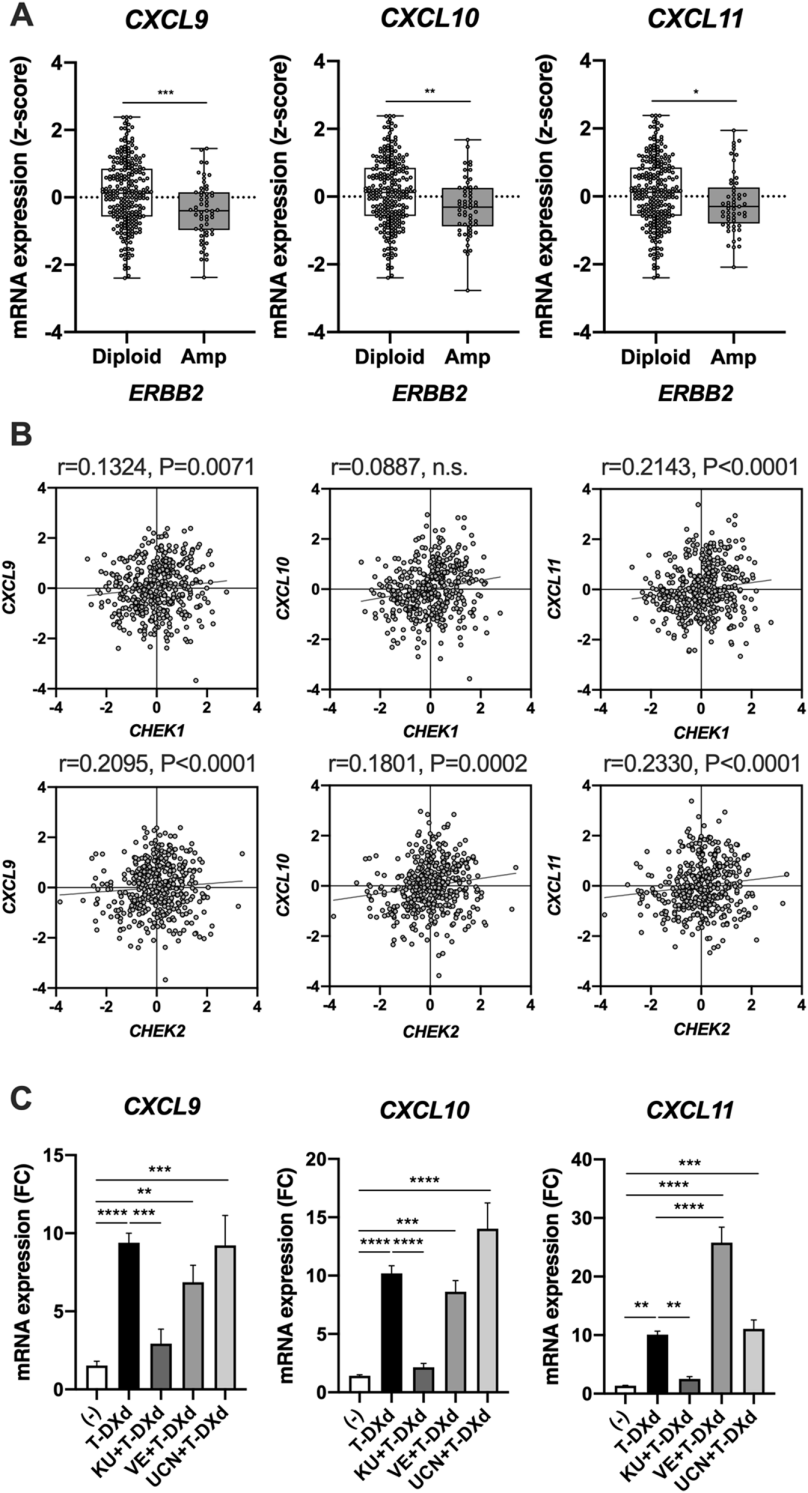
Involvement of DNA damage signaling in the induction of mRNA expression of *CXCL9/10/11* by T-DXd in HER2-positive GC cells. (**A**) Comparison of mRNA expression (z-score) of *CXCL9*, *CXCL10*, and *CXCL11* between *ERBB2* amplified GC tissues (Amp) (n = 55) and ERBB2 non-amplified GC tissues (Diploid) (n = 238) from TCGA dataset. (**B**) Correlation among mRNA expression (z-score) of *CXCL9*, *CXCL10*, *CXCL11*, *CHEK1*, *CHEK2*, and *TP53* in samples from 412 patients with GC from TCGA dataset. (**C**) qPCR analysis of *CXCL9, CXCL10,* and *CXCL11* in NCI-N87 cells treated with 1 μg/ml T-DXd in the absence or presence of 10 μM KU-55933 (KU), 10 μM VE-821 (VE), or 100 nM UCN-01 (UCN) for 72 h (n = 3). Values are shown as means ± SEM. *P < 0.05, **P < 0.01, ****P < 0.0001.

We also investigated the underlying mechanism of how T-DXd up-regulated mRNA expression of *CXCL9/10/11* in HER2-positive GC cells. Because trastuzumab slightly increased mRNA expression of *CXCL9/10/11* in HER2-positive GC cells (Fig. 3C), there is probably a link between HER2 activity and the expression of *CXCL9/10/11*. We, therefore, examined the association between HER2 level and CXCL*9/10/11* expression in GC tissues. TCGA dataset showed that mRNA expression of *CXCL9/10/11* was decreased in HER2-amplified GC tissues as compared with that in HER2-non amplified GC tissues (Fig. 4A), suggesting that mRNA expression of *CXCL9/10/11* might be inversely associated with the activity of HER2 signaling in GC. However, our present data suggest that T-DXd has little effect on the downstream signaling of HER2 including Akt and ERK pathways (Fig. 2B). Thus, additional mechanisms must exist to up-regulate *CXCL9/10/11* by T-DXd. Previous reports suggested that anti-cancer drugs such as cisplatin and hydroxyurea induced mRNA expression of *CXCL9/10/11* through cell cycle-specific DNA damage in human cancer cells^26,27^, and the DNA topoisomerase I inhibitor, irinotecan, is also known to cause cell cycle-specific DNA damage^16,30^. Indeed, we found that irinotecan significantly induced mRNA expression of *CXCL9/10/11* in NCI-N87 cells (Supplementary Fig. S4). Because DNA damage-induced checkpoint activation is partially regulated by ataxia telangiectasia-and-rad3-related (ATR) and ataxia-telangiectasia mutated (ATM) pathways^31^, we also examined the involvement of DNA damage signalings such as ATR and ATM pathways in the induction of mRNA expression of *CXCL9/10/11* by T-DXd in HER2-positive GC cells. As a result, we found that weak but significant correlations exist between mRNA expression of the signaling molecules involved in ATR and ATM pathways such as chk1 (*CHEK1*) and chk2 (*CHEK2*), and chemokines *CXCL9/10/11* in GC tissues (Fig. 4B). We also demonstrated that T-DXd-triggered mRNA expression of *CXCL9/10/11* was significantly attenuated by an ATM inhibitor (KU), but not ATR inhibitor (VE) and chk1 inhibitor (UCN) in HER2-positive GC cells (Fig. 4C), suggesting that T-DXd increased mRNA expression of *CXCL9/10/11* through the activation of ATM- but not ATR/chk1-mediated DNA damage signaling pathway in HER2-positive GC cells. These results suggest that T-DXd might increase mRNA expression of *CXCL9/10/11* through DXd-mediated activation of DNA damage signaling in HER2-positive GC cells.

**Figure 5:**
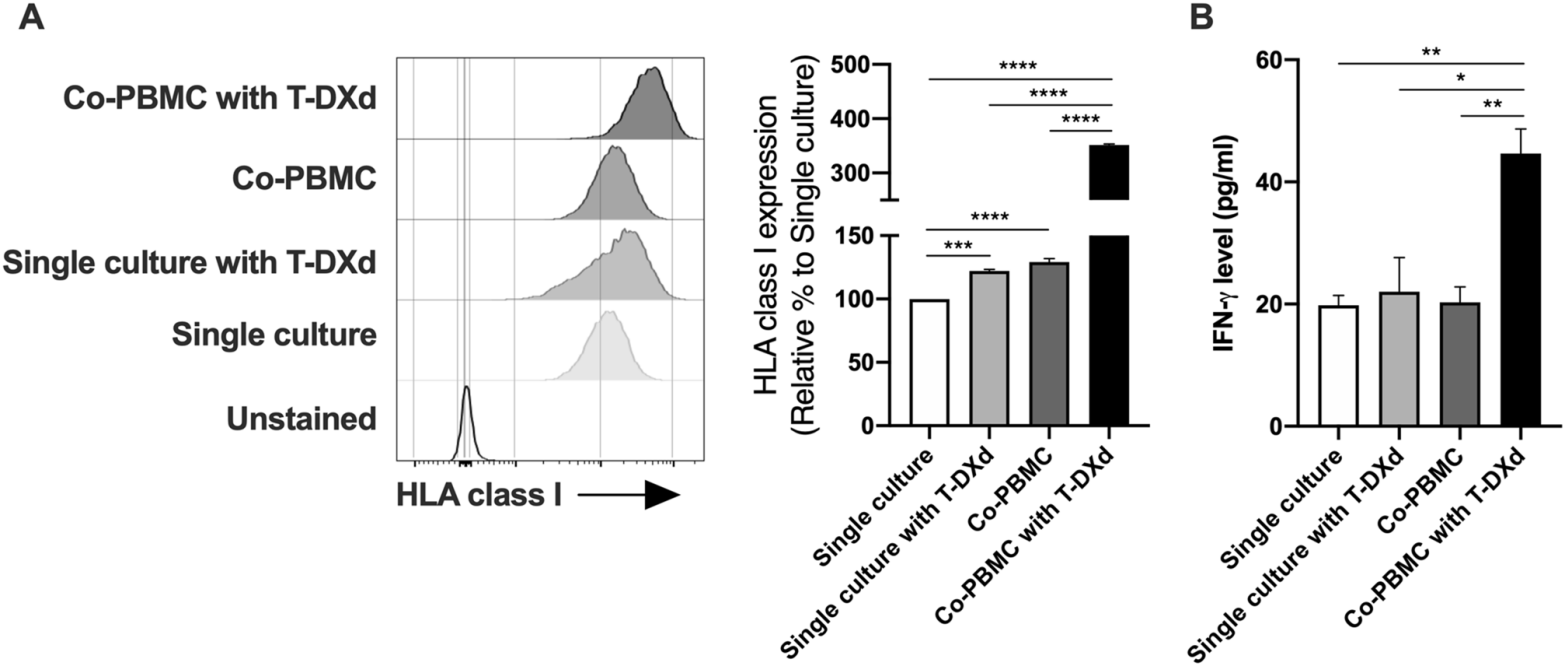
The effect of T-DXd on the expression of HLA class I in HER2-positive GC cells co-cultured with PBMC. (**A**) The expression of HLA class I in NCI-N87 cells co-cultured with or without PBMC in the absence or presence of 1 μg/ml T-DXd for 72 h (n = 3). Single culture, single culture of NCI-N87 cells; Co-PBMC, NCI-N87 cells co-cultured with PBMC. Representative histograms were shown. (**B**) IFN-γ production in supernatants of NCI-N87 co-cultured with or without PBMC in the absence or presence of T-DXd for 72 h (n = 3). Values are shown as means ± SEM. *P < 0.05, ***P < 0.001, ****P < 0.0001.

Because T-DXd increased the expression of CXCL9/10/11 in HER2-positive GC cells, recruited immune cells by the chemokines might also affect HLA class I expression on GC cells through the production of cytokines such as IFN-γ. Therefore, we finally examined the effect of immune cells including T cells on HLA class I expression of T-DXd-treated HER2-positive GC cells. NCI-N87 cells were co-cultured with or without human peripheral blood mononuclear cells (PBMC) in the absence or presence of T-DXd and subjected to flow cytometry to analyze the expression of HLA class I on NCI-N87 cells. The cell surface expression of HLA class I on NCI-N87 cells was slightly increased by the treatment with T-DXd or the co-culture with PBMC, while T-DXd dramatically enhanced the expression of HLA class I on NCI-N87 cells co-cultured with PBMC (Fig. 5A), suggesting that immune cells including T cells further enhance the expression of HLA class I on HER2-positive GC cells in the presence of T-DXd. We also found that the level of IFN-γ production was not changed in NCI-N87 cells by the treatment with T-DXd or co-cultured with PBMC, while it was significantly increased in NCI-N87 cells co-cultured with PBMC in the presence of T-DXd (Fig. 5B). This result suggests that IFN-γ might be one of the crucial factors that is involved in the up-regulation of HLA class I expression in NCI-N87 cells co-cultured with PBMC in the presence of T-DXd.

Taken together, our data suggest that T-DXd slightly enhances the expression of HLA class I in HER2-positive GC cells, and it also increases the expression of *CXCL9/10/11* through the activation of ATM-mediated DNA damage signaling pathway in HER2-positive GC cells.

## Discussion

In the current study, we identify for the first time the effect of T-DXd on the expressions of HLA class I and chemokines, CXCL9/10/11, in HER2-positive GC cells. Although T-DXd slightly up-regulated HLA class I expression, it markedly increased mRNA expression of *CXCL9/10/11* in HER2-positive GC cells. We also found that T-DXd-induced mRNA expression of *CXCL9/10/11* was mediated through the activation of the DNA damage signaling pathway regulated by ATM and not ATR or Chk1 in HER2-positive GC cells.

We found that T-DXd inhibited cell proliferation of not only trastuzumab-sensitive HER2-positive GC cells (NCI-N87 and OE19 cells) but also trastuzumab-resistant HER2-positive GC cells (MKN7 cells) (Fig. 1C). Because trastuzumab markedly decreased the expression of HER2 in MKN7 cells (Fig. 2 and Supplementary Fig. S1), downregulated HER2 expression and lower binding of trastuzumab to MKN7 cells might be the main mechanism of resistance to trastuzumab. However, T-DXd significantly suppressed cell proliferation of MKN7 cells even in such trastuzumab-resistant HER-2 positive GC cells. Indeed, Ogitani et al. revealed that T-DXd has the potential to deliver sufficient DXd into cancer cells regardless of HER2 levels, that is, T-DXd can inhibit cell proliferation of not only HER2-strong positive cells but also HER2-weak positive cells. Therefore, T-DXd significantly suppressed cell proliferation of HER2-downregulated MKN7 cells^1^, which is consistent with the previous report that T-DXd has a clinical benefit for HER2-positive patients who had failed to trastuzumab therapy^5^.

In the present study, moderately increased expression of HLA class I by T-DXd was observed in HER2-positive GC cells. Although we evaluated the effect of T-DXd on the expression of HLA class I in HER2-positive GC cells by both flow cytometry and western blot analyses, we could only detect a slight increase in cell surface expression of HLA class I (Fig. 2). On the other hand, Iwata et al. demonstrated that T-DXd significantly enhanced murine MHC class I expression on tumors in an in vivo xenograft mouse model with human HER2-expressing murine CRC cells^6^. They also showed that DXd increased the expression of MHC class I in murine CRC cells in vitro, suggesting that T-DXd could directly trigger the expression of MHC class I in HER2-positive cancer cells. The discrepancy between the previous and present findings is unclear. The regulation of MHC class I expression in HER2-positive cancer cells by T-DXd may depend on the cell type (e.g., CRC cells vs. GC cell lines, species differences [mice and human]) and methods of stimulation and analysis (e.g., T-DXd vs. DXd, treatment duration, and assay time, etc.).

At present, molecular mechanisms by which T-DXd up-regulates cell surface expression of HER2 in NCI-N87 and OE19 cells are largely unknown. Because T-DXd and irinotecan but not trastuzumab significantly increased HER2 expression (Fig. 2A, Supplementary Figs. S1, S2), the cytotoxic effect of topoisomerase I inhibitor such as DNA strand breaks might be involved in the up-regulation of cell surface expression of HER2 in NCI-N87 and OE19 cells. ATM kinase plays a central role in sensing DNA double-stranded breaks and coordinating their repair, and DXd activates DNA-damage response pathways including ATM pathway^1, 28^. Interestingly, a recent report suggested that ATM functions as a modulator of HER2 receptor levels and stability. Indeed, suppression of ATM kinase activity by a specific inhibitor significantly reduced HER2 receptor levels in vivo^32^. Therefore, T-DXd possibly up-regulates cell surface HER2 levels in NCI-N87 and OE19 cells through the activation of the ATM pathway.

Our data suggest that the ATM-mediated DNA damage signaling pathway might be involved in T-DXd-induced mRNA expression of *CXCL9/10/11* in HER2-positive GC cells, because an ATM inhibitor (KU-55933), but not ATR and Chk1 inhibitors (VE-821 and UCN-01), selectively inhibited T-DXd-induced mRNA expression of *CXCL9/10/11* (Fig. 4B). ATM and its downstream molecule Chk2 are the primary kinases responsible for G1/S phase arrest of the cell cycle^33,34^, and S-phase-specific DNA damage has been reported to activate the immune response including induction of CXCL9, -10 expression in cancer cells^26,27^. T-DXd contains the DNA topoisomerase I inhibitor (DXd), and DNA topoisomerase I inhibitors such as irinotecan act on the S and G2 phases of the cell cycles^16,30^. Therefore, T-DXd especially DXd might cause S-phase-specific DNA damage and consequently trigger mRNA expression of *CXCL9/10/11* in HER2-positive GC cells.

In summary, our present study revealed that T-DXd increased surface expression of HLA class I and up-regulated T cell chemoattractants, CXCL9/10/11, in HER2-positive GC cells. These results suggest a possibility that CXCL9/10/11 released from HER2-positive GC cells by T-DXd might attract tumor-infiltrating lymphocytes, which produce a large amount of IFN-γ^35,36^, leading to activation of immune cells and further enhancement of HLA class I expression on cancer cells (Fig. 5 and Supplementary Fig. S5). Therefore, T-DXd might enhance anti-tumor immune responses in human GC. However, in the current study, we performed only in vitro experiments. Further investigations using GC tissues from clinical trials of T-DXd are required.

## Methods

### Materials

T-DXd was provided by Daiichi Sankyo Co., Ltd. (Tokyo, Japan). Other reagents used in this study were acquired from the indicated suppliers: trastuzumab (Chugai Pharmaceutical Corporation, Tokyo, Japan); KU-55933, VE-821, and UCN-01 (Merck Sigma-Aldrich, St. Louis, MO, USA); IFN-γ (R&D systems, Minneapolis, MN, USA); irinotecan (FUJIFILM Wako Pure Chemical Corporation, Osaka, Japan); ultra-LEAF purified human IgG1 isotype control recombinant antibody (BioLegend, San Diego, CA, USA); FITC mouse anti-human HER-2/neu (340553), FITC mouse IgG1 κ isotype control (555909), and 7-AAD (559925) (BD Biosciences, San Jose, CA, USA); PE anti-human HLA-ABC monoclonal antibody (W6/32) (12-9983-42), Alexa Fluor 488 anti-human CD326 (EpCAM) (53-8326-41), and PE mouse IgG2a κ isotype control (eBM2a) (12-4724-81) (Thermo Fisher Scientific, Waltham, MA, USA); HER2/ErbB2 rabbit mAb (#4290), Phospho-Akt rabbit mAb(#4060), Akt (pan) rabbit mAb (#4691), Phospho-ERK1/2 rabbit mAb (#4370), and ERK1/2 rabbit mAb (#4695) (Cell Signaling Technology, Danvers, MA, USA); anti-human HLA class I (HLA-ABC) mAb (D367-3) (Medical & Biological Laboratory, Aichi, Japan); Anti-β-actin antibody (sc-69879) (Santa Cruz Biotechnology, Dallas, TX, USA).

### GC cell lines

The human GC cell lines were purchased from the indicated suppliers: NCI-N87 and AGS (the American Type Culture Collection, Manassas, VA, USA); MKN7 and NUGC3 (the Japanese Collection of Research Bioresources Cell Bank, Osaka, Japan); OE19 (the European Collection of Animal Cell Cultures, Salisbury, UK). The cells were maintained in RPMI-1640 (Sigma-Aldrich) supplemented with 10% heat-inactivated FBS and penicillin/streptomycin in a 5% CO_2_ atmosphere at 37 ℃. The information regarding genetic alterations and mutations in these cell lines was based on cBioportal database.

### Cell proliferation assay

Cells were seeded at 1000 cells/well in a 96-well plate and treated with different concentrations of IgG, Herceptin, or T-DXd for 6 days. The cell viability of each cell line was determined by the Cell Counting Kit-8 (Dojindo Molecular Technologies, Kumamoto, Japan).

### Flow cytometry analysis

Cells were stained with antibodies specific for PE anti-human HLA class I and FITC anti-human HER2. After washing with PBS, the stained cells were incubated with 7-AAD in 1× binding buffer for 10 min at room temperature and analyzed on a BD FACSCanto II flow cytometer (BD Biosciences). Flow cytometry data were analyzed using FlowJo 10.7.1 (FlowJo, Ashland, OR, USA, proprietary commercial software, https://www.flowjo.com/).

### Western blot analysis

Cells were lysed in RIPA Lysis and Extraction Buffer (Thermo Fisher Scientific) with protease inhibitor cocktail (Thermo Fisher Scientific) and phosphatase inhibitor (FUJIFILM Wako Pure Chemical Corporation). The cell lysate was dissolved in sample buffer (G-Biosciences, Louis, MO, USA) containing 50 mM dithiothreitol, and then boiled for 10 min. Protein concentrations were measured on a NanoDrop ND-1000, and 10 μg proteins were subjected to SDS-page gels and transferred to polyvinylidene fluoride membranes. Blots were immersed in 5% milk blocking solution for 1 h at room temperature, followed by incubation with primary antibody solution overnight at 4 ℃. Membranes were washed three times with TBS/T and then incubated in a secondary antibody solution for 2 h at room temperature. Immunoreactive proteins were visualized using ECL prime (GE Healthcare, Chicago, IL, USA) followed by image analysis (LAS-4000 mini, Fuji Film, Tokyo, Japan).

### Quantitative real-time PCR

Total RNA was isolated from GC cell lines using the TRIzol reagent (QIAGEN, Valencia, CA, USA). RNA was quantified on a NanoDrop ND-1000 spectrophotometer (Thermo Fisher Scientific). Complementary DNA (cDNA) was synthesized using the PrimeScript RT-PCR Kit (Takara, Otsu, Japan). Quantitative real-time PCR (qPCR) was performed on a QuantStudio 3 Fast Real-Time PCR System (Applied Biosystems, Carlsbad, CA, USA) using PrimeTime Gene Expression Master Mix (Integrated DNA Technologies, Coralville, IA, USA) with specific primers and probes against human *CXCL9* (NM_002416), *CXCL10* (NM_001565), *CXCL11* (NM_005409), and *GAPDH* (NM_002046) (Integrated DNA Technologies). qPCR data were normalized against the corresponding levels of *GAPDH* mRNA.

### The Cancer Genome Atlas (TCGA) dataset analysis

The mRNA expression z-score of genes (RNA-Seq V2 RSEM normalized, RNA-Seq data) were obtained from TCGA stomach adenocarcinoma dataset (PanCancer Atlas) (n = 440) through cBioPortal^37^. The correlations between mRNA expressions of indicated molecules in the Figures of this study in TCGA cohorts were analyzed.

### Co-culture of HER2-positive GC cells with PBMC

NCI-N87 cells were co-cultured with or without PBMC in the absence or presence of 1 μg/ml T-DXd for 72 h. After 72 h of incubation, the cells were stained with antibodies specific for PE anti-human HLA-ABC antibody and Alexa Fluor 488 anti-human CD326 (EpCAM) antibody. After washing with PBS, the stained cells were incubated with 7-AAD in 1× binding buffer for 10 min at room temperature and analyzed the expression of HLA class I in NCI-N87 cells (gated on CD326^+^ cells) by a BD FACSCanto II flow cytometer (BD Biosciences).

### Enzyme-linked immunosorbent assay (ELISA)

The concentration of IFN-γ in supernatants of cell cultures was determined by ELISA. The kit for human IFN-γ (DIF50C) was obtained from R&D systems.

### Statistical analysis

Data are expressed as means ± SEM. The statistical analyses were performed using Graph pad prism 8.4.3 (GraphPad, San Diego, CA, USA, proprietary commercial software, https://www.graphpad.com/). For two-group comparisons, statistical analyses were performed using the unpaired t-test. For multigroup comparisons, we applied one-way ANOVA with post hoc Tukey–Kramer test. The Spearman correlation test was used to analyze the association in each experiment. A value of *p* < 0.05 was considered to be significant.

## Supplementary Information

Supplementary Information.

## Abbreviations

ATM: Ataxia-telangiectasia mutated
ATR: Ataxia telangiectasia-and-rad3-related
CXCL: C–X–C motif chemokine ligand
GC: Gastric cancer
HER2: Human epidermal growth factor-2
HLA: Human leukocyte antigen
IFN-γ: Interferon-γ
MHC: Major-histocompatibility-complex
TCGA: The Cancer Genome Atlas
T-DXd: Trastuzumab deruxtecan
