# Supplementary material for: The effects of T-DXd on the expression of HLA class I and chemokines CXCL9/10/11 in HER2-overexpressing gastric cancer cells
Source: Sci Rep. 2021 Aug 19;11:16891. doi: 10.1038/s41598-021-96521-2 (PMC8376901; doi:10.1038/s41598-021-96521-2)

Original article

The effects of T-DXd on the expression of HLA class I and chemokines CXCL9/10/11 in HER2-overexpressing gastric cancer cells

Shotaro Nakajima,[1,2] Kosaku Mimura,[1,3] Takuro Matsumoto,[1] Aung Kyi Thar Min,[1] Misato Ito,[1] Hiroshi Nakano,[1] Prajwal Neupane,[1] Yasuyuki Kanke,[1] Hirokazu Okayama,[1] Motonobu Saito,[1] Tomoyuki Momma,[1] Yohei Watanabe,[1] Hiroyuki Hanayama,[1] Suguru Hayase,[1] Zenichiro Saze,[1] and Koji Kono[1,†]

[1]*Department of Gastrointestinal Tract Surgery,*

[2]*Department of Obesity and Inflammation Research,*

[3]*Department of Blood Transfusion and Transplantation Immunology,*

Fukushima Medical University School of Medicine, Fukushima, Japan

[†]**Corresponding author: Koji Kono**

Department of Gastrointestinal Tract Surgery, Fukushima Medical University School of Medicine,

1 Hikariga-oka, Fukushima city, Fukushima 960-1295, Japan

Tel.:+81-24-547-1259; Fax.: +81-24-547-1980

E-mail: kojikono@fmu.ac.jp

Running title: The effect of T-DXd on HER2-positive GC

Supplementary Figure Legends

Supplementary Figure S1. The effect of trastuzumab on the expression of HLA class I in HER2-positive GC cells.

(A) Cell surface expression of HER2 (*left*) and HLA class I (*right*) in HER2-positive GC cell lines treated with several concentrations of trastuzumab or 100 ng/ml IFN-γ for 72 h (n=3). Representative histograms were shown. Values are shown as means ± SEM. *P <0.05, **P <0.01, ***P <0.001, ****P <0.0001. (B) Western blot analysis of the indicated molecules in HER2-positive GC cell lines treated with several concentrations of trastuzumab for 72 h.Values are shown as means ± SEM. *P <0.05, ***P <0.001, ****P <0.0001.

Supplementary Figure S2. The effect of irinotecan on the expression of HLA class I in HER2-positive GC cells.

Cell surface expression of HER2 and HLA class I in HER2-positive GC cell lines treated with several concentrations of trastuzumab or 100 ng/ml IFN-γ for 72 h (n=3). Representative histograms were shown. Values are shown as means ± SEM. ***P <0.001, ****P <0.0001.

Supplementary Figure S3. The effect of T-DXd on the expression of HLA class I in HER2-negative GC cells.

The expression of HER2 (*left*) and HLA class I (*right*) in HER2-negative GC cell lines treated with several concentrations of T-DXd or 100 ng/ml IFN-γ for 72 h (n=3). Representative histograms were shown. Values are shown as means ± SEM. *P <0.05, ***P <0.001, ****P <0.0001.

Supplementary Figure S4. The effect of irinotecan on mRNA expression of *CXCL9/10/11* in HER2-positive GC cells.

mRNA expression of *CXCL9*, *CXCL10,* and *CXCL11* in NCI-N87 cells treated with or without 10 or 50 μM irinotecan for 72 h (n=3). Values are shown as means ± SEM. ***P* <0.05, ****P* <0.001.

Supplementary Figure S5. Schematic representation of the effect of T-DXd on GC.

T-DXd moderately increases HLA class I expression in HER2-positive GC cells. T-DXd also triggers the expression of CXCL9/10/11 in HER2-positive GC cells, which might recruit tumor-infiltrating lymphocytes into GC tissues. CXCL; C-X-C motif chemokine ligand, GC; gastric cancer, HER2; human epidermal growth factor-2, HLA; human leukocyte antigen, IFN-γ; interferon-γ, T-DXd; trastuzumab deruxtecan, TIL; tumor-infiltrating lymphocytes

Supplementary Figure S6. Full-length blots shown in figures and supplementary figures.

Supplementary Figure S1

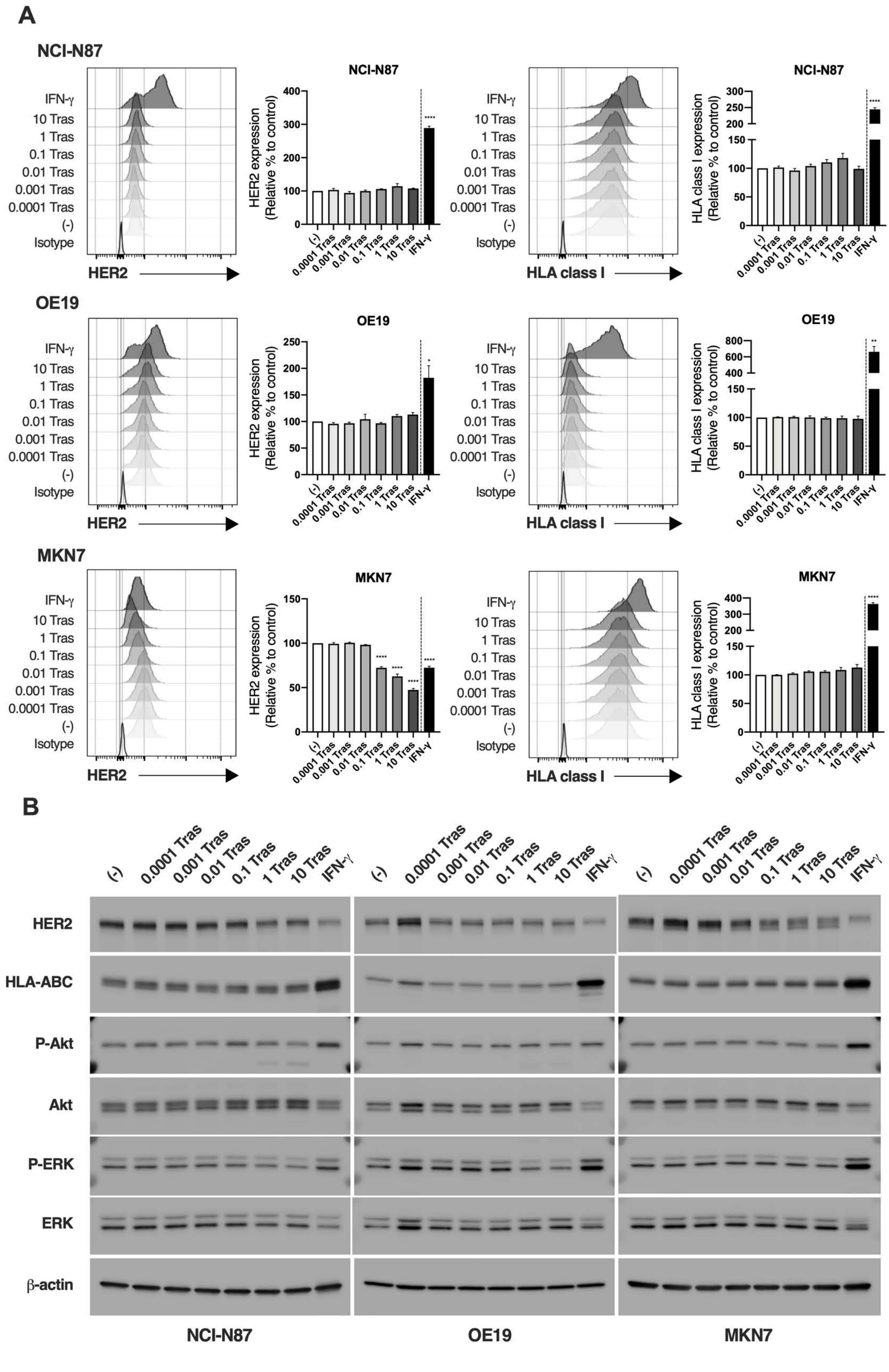

Supplementary Figure S2

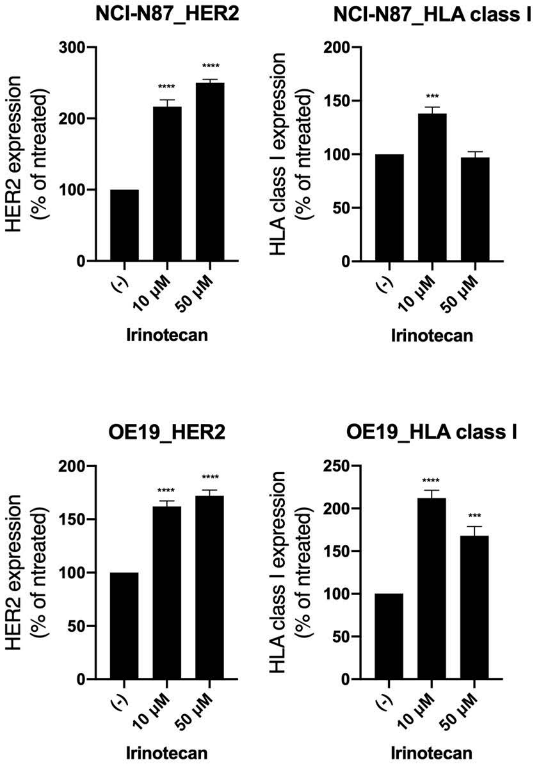

Supplementary Figure S3

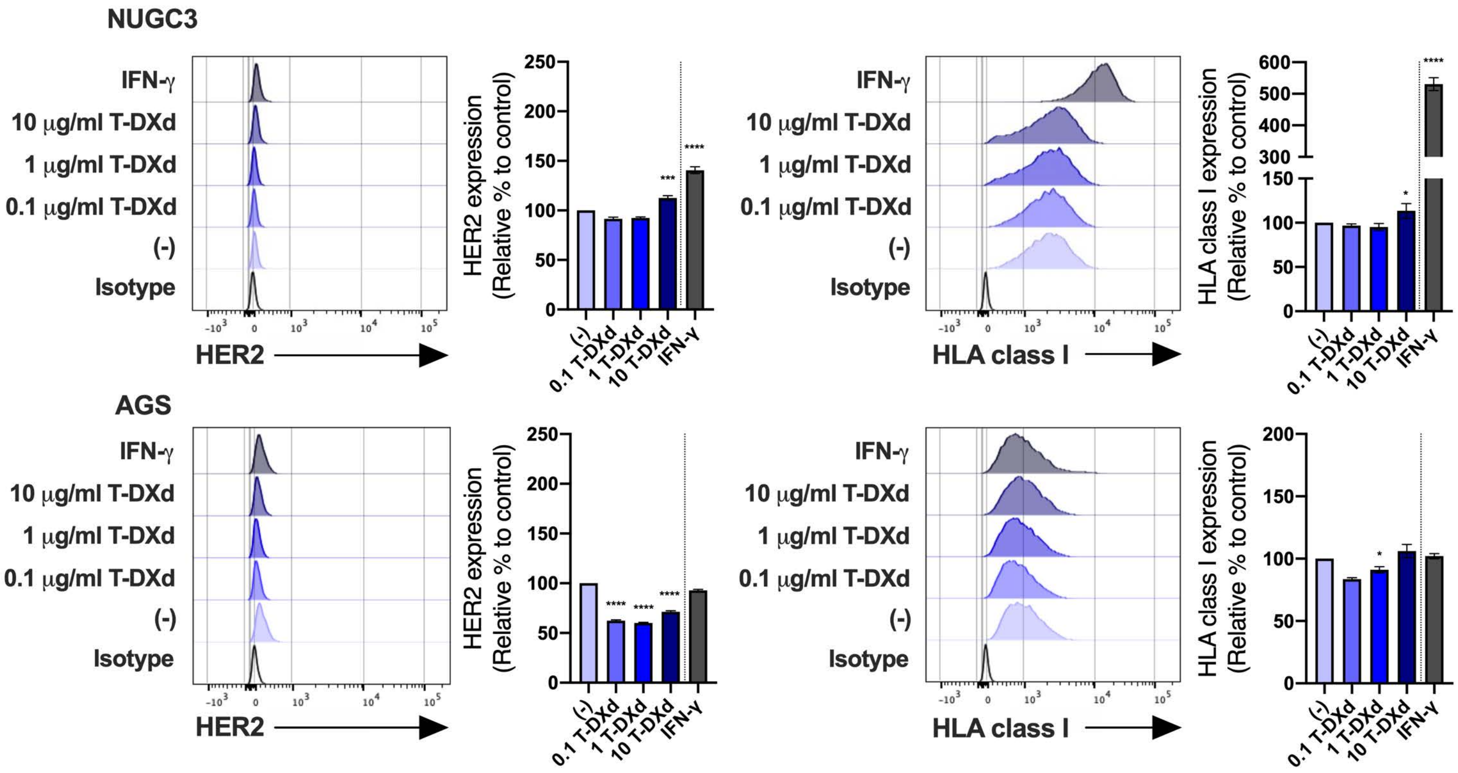

Supplementary Figure S4

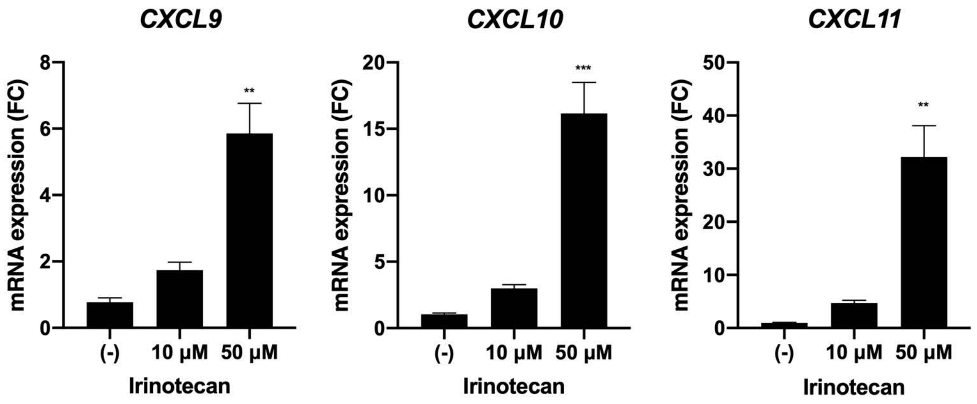

Supplementary Figure S5

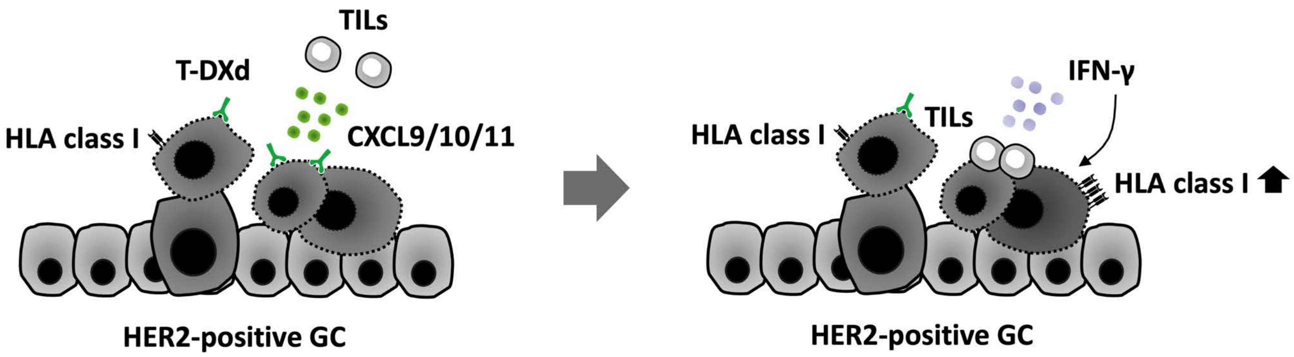

Supplementary Figure S6-1

Fig. 2B_NCI-N87

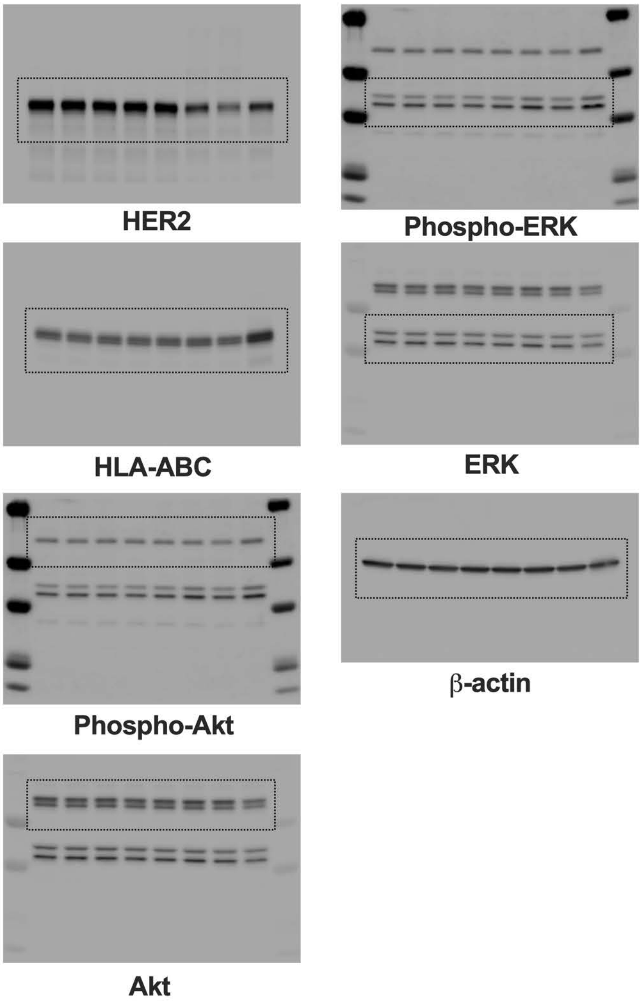

Fig. 2B_OE19

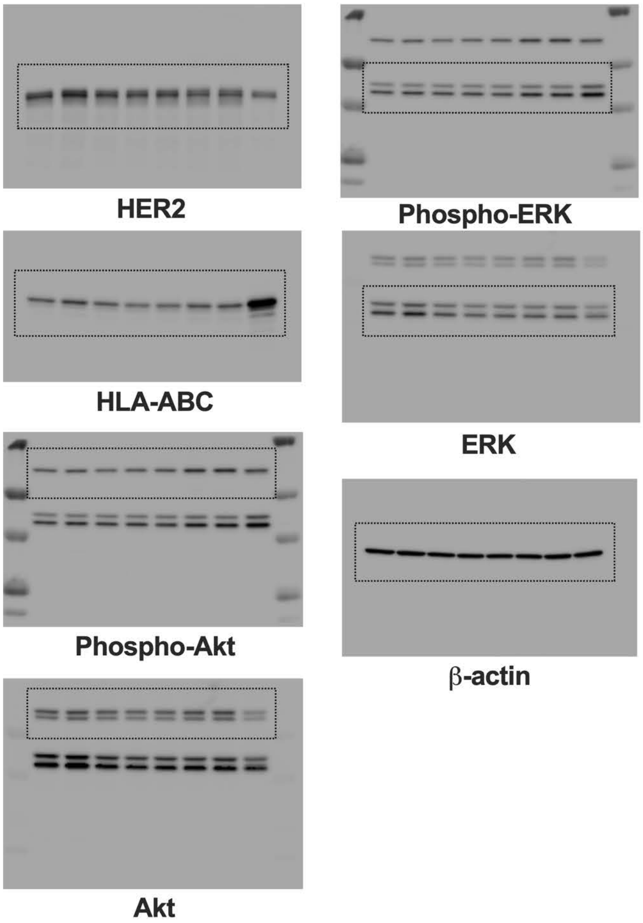

Fig. 2B_MKN7

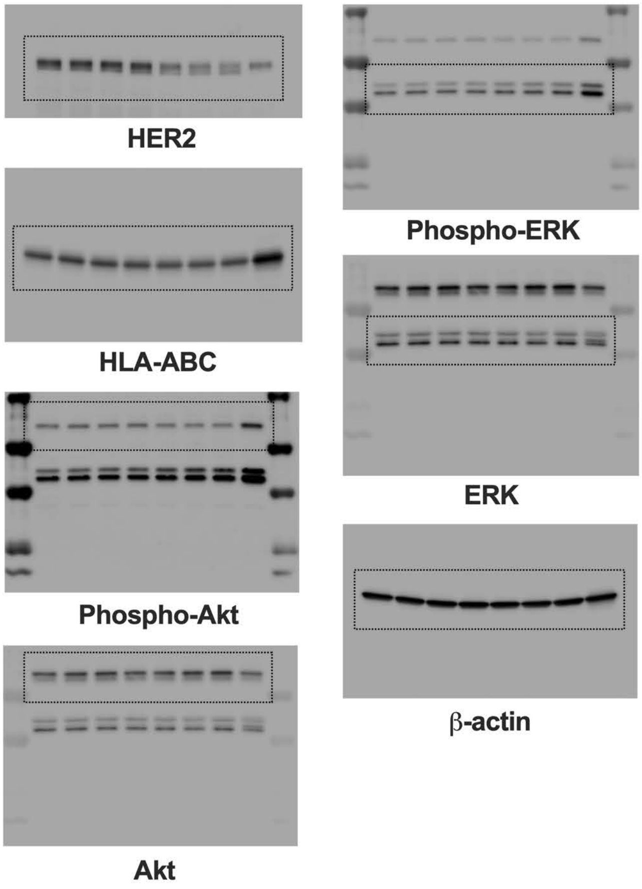

Supplementary Figure S6-2

Supplementary Fig. S1B_NCI-N87

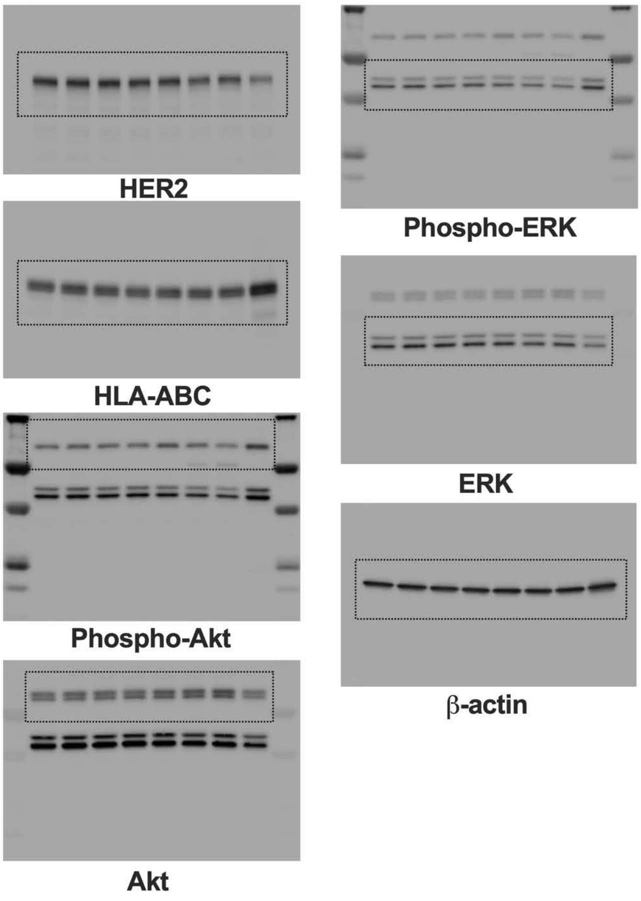

Supplementary Fig. S1B_OE19

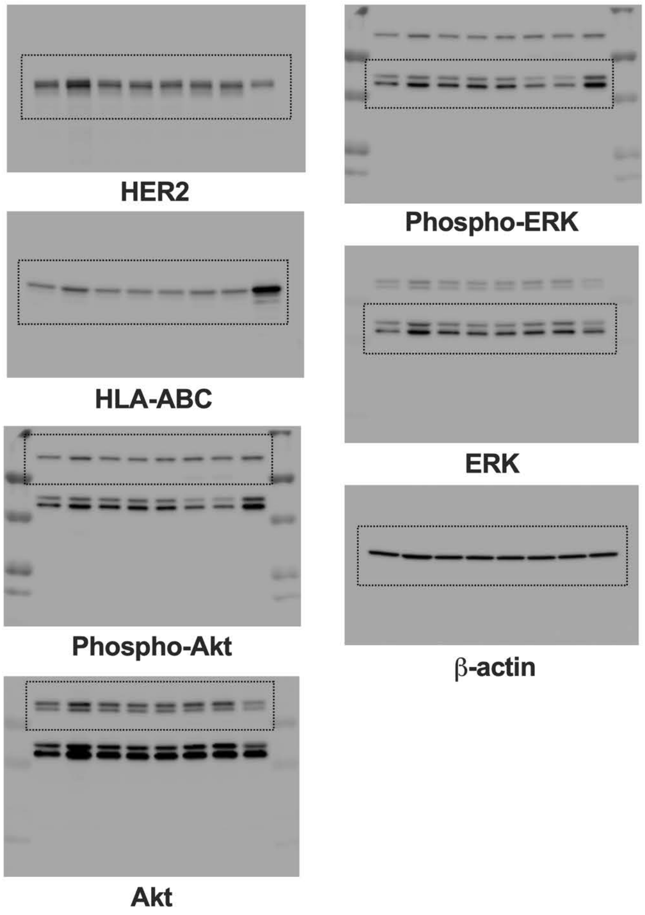

Supplementary Fig. S1B_MKN7

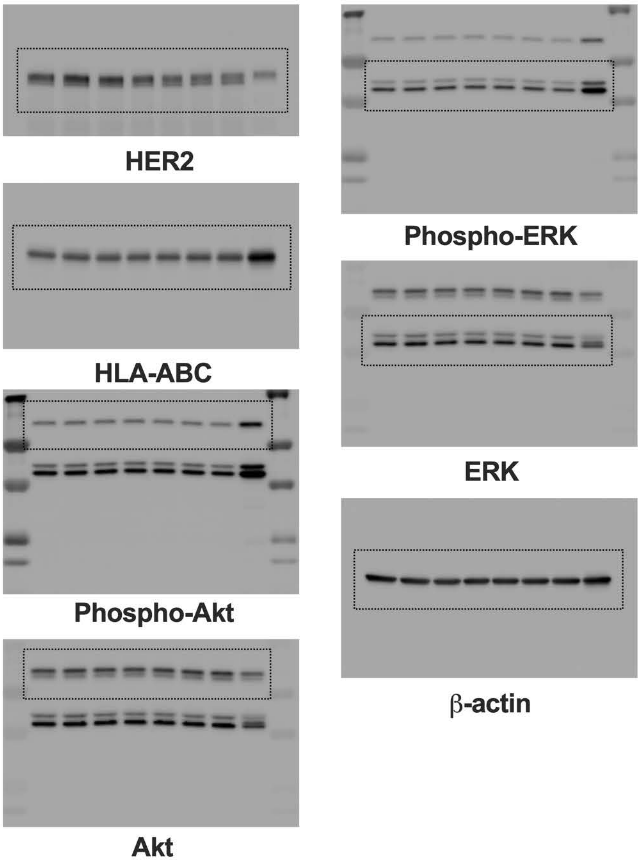

Supplement: Supplementary file 1 — Supplementary Information. [file 41598_2021_96521_MOESM1_ESM.pdf]
